# Whole body potassium as a biomarker for potassium uptake using a mouse model

**DOI:** 10.1038/s41598-021-85233-2

**Published:** 2021-03-18

**Authors:** Sana Tabbassum, Pinjing Cheng, Frank M. Yanko, Rekha Balachandran, Michael Aschner, Aaron B. Bowman, Linda H. Nie

**Affiliations:** 1 School of Health Sciences, Purdue University, West Lafayette, 47906 USA; 2 School of Nuclear Science and Technology, University of South China, Hengyang, China; 3 Department of Molecular Pharmacology, Albert Einstein College of Medicine, Bronx, NY 10461 USA

**Keywords:** Biophysics, Developmental biology, Physiology, Biomarkers, Medical research, Engineering, Physics

## Abstract

Potassium is known for its effect on modifiable chronic diseases like hypertension, cardiac disease, diabetes (type-2), and bone health. In this study, a new method, neutron generator based neutron activation analysis (NAA), was utilized to measure potassium (K) in mouse carcasses. A DD110 neutron generator based NAA assembly was used for irradiation.Thirty-two postmortem mice (n= 16 males and 16 females, average weight $22.02\pm 1.3$ and $17.9\pm 1.1$ g) were employed for this study. Soft-tissue equivalent mouse phantoms were prepared for the calibration. All mice were irradiated for 10 minutes, and the gamma spectrum with 42K was collected using a high efficiency, high purity germanium (HPGe) detector. A lead shielding assembly was designed and developed around the HPGe detector to obtain an improved detection limit. Each mouse sample was irradiated and measured twice to reduce uncertainty. The average potassium concentration was found to be significantly higher in males $(2846 \pm 525 \upmu g/g)$ compared to females $(2116.2 \pm 432 \upmu g/g)$. We also observed a significant correlation between potassium concentration and the weight of the mice. The detection limit for potassium quantification with the NAA system was 46 ppm. The radiation dose to the mouse was approximately 56 ${ \pm 1.6 }$ mSv for 10-min irradiation. In conclusion, this method is suitable for estimating individual potassium concentration in small animals. The direct evaluation of total body potassium in small animals provides a new way to estimate potassium uptake in animal models. This method can be adapted later to quantify potassium in the human hand and small animals in vivo. When used in vivo, it is also expected to be a valuable tool for longitudinal assessment, kinetics, and health outcomes.

## Introduction

Almost half of adults in the United States (108 million, or 45%) have hypertension—one of the well-known risk factors for heart attack and stroke^1^. Many clinical trials and epidemiological and intervention studies support the finding that blood pressure and associated risks decrease significantly by lowering sodium (Na) and increasing the K intake^2–6^. Potassium is the most abundant intracellular cation that plays a critical role in transmitting nerve impulse, cardiac activity, membrane transport, acid–base balance, and neuromuscular functions^7^. Numerous scientific and clinical studies showed that potassium-rich diets such as DASH [Dietary Approaches to Stop Hypertension] has successfully helped people to achieve lower blood pressure and overall decreased kidney disease progression^6,8–10^. As the DASH diet is rich in other micro-nutrients as well, potassium is not unequivocally accepted as a significant benefiting component^11^. Numerous studies have been conducted on the association of potassium and blood pressure association; however, insufficient data is available to support the hypothesis that potassium is an actual mediator of blood pressure^5,9,11,12^. The Agency for Healthcare Research and Quality (AHRQ) and DRI 2019 committee identified the limitations of current potassium balance studies and concluded that evidence is insufficient to estimate the potassium’s adequacy requirement. This implies that studies available in literature lack rigor and consistency to draw concrete conclusions about potassium^13,14^. Likewise, it is also not very clear how higher or lower potassium intake alone or in the presence of other nutrients can prevent high blood pressure (BP) and cardiovascular diseases (CVD). The AHRQ committee acknowledged the relationship between potassium and reduction in blood pressure but concluded that the existing studies were insufficient to establish a potassium Chronic Disease Risk Reduction Intake (CDRRI)^14^. Hence, there is a dire need to synthesize the evidence that increasing potassium intake itself helps to prevent hypertension, stroke, kidney stones, cardiovascular diseases, and associated morbidity and mortality risks. In light of the AHRQ and 2019 DRI committee’s recommendation, more evidential based research is required to determine adequate intake level that will help reduce the disease (e.g., BP-CVD, Calcium retention-Osteoporotic fracture) and mortality risk^13,14^. The nutrition studies and clinical recommendations for dietary intake rely on potassium measurement through serum and urine. Although recovery biomarker, i.e., 24 h urine collection, is considered the gold standard to estimate the potassium balance^6,15^, recently simulated space flight studies showed that a single 24-h urine collection could not predict sodium, potassium, or chloride intake^16^. Therefore, to reduce day-to-day basis variation and incomplete urine collection rate ( 40%), multiple samples are required, which increases the inconvenience and cost^16–18^. Additionally, recovery biomarkers cannot be beneficial when diuretics, alterations in acid-base balance, or chronic kidney diseases disrupt the neutral balance between intake and potassium excretion^19^. On the other hand, few MRI based studies found a correlation of tissue sodium with hypertension, type 2 diabetes Miletus, and acute heart failure^20–22^. They hypothesized that intracellular sodium accumulation in muscle happened at the expense of muscle potassium^20^. Nevertheless, no evidential data about similar potassium studies are available, and clearly, the long term health impact of such decreased potassium stores are understudied and not well understood. New studies regarding whole-body potassium retention, adequate intake levels, the association between dietary potassium, and potential health outcomes are required to fill the existing gaps of knowledge on potassium and health. However, technological hurdles have been a barrier to such studies. Here we sought to determine if the application of neutron generator based NAA technology may provide a valuable tool in this line of research.The in vivo neutron activation analysis (IVNAA) is a promising technique commonly employed to quantify the total content of essential elements such as nitrogen calcium, manganese, aluminum, and sodium in the human body^23,24^. The IVNAA technique gained attention in the field of nutritional and metabolic studies since the 1970s^24^. However, little work was done on assessing the kinetic behavior of elements like sodium and potassium, which are closely associated with many health issues^25^. Potassium (K), an essential intracellular electrolyte in the human body, has been mostly investigated in terms of total body concentration only^26^. The in vivo total body potassium (TBK) has been measured using whole-body counters and occasionally with neutron activation analysis^27^. Nevertheless, in vivo potassium distribution, turnover rate, and metabolism activity studies are sparse to the best of our knowledge. The large accelerator-based irradiation facilities were most commonly used for clinical applications of in vivo neutron activation analysis^23^. Contrary to these bulky and expensive accelerator-based setups, our lab has developed a sensitive and compact, deuterium–deuterium (DD) neutron generator based irradiation system^28,29^. Our IVNAA system has an approximate fluence of $1 X 10^9$ n/s, surrounded by a customized moderator- reflector assembly. This system is transportable and provides a comparable sensitivity and lower dose^28,29^. Our two main objectives for this study were to investigate the feasibility of using the in vivo neutron activation analysis (IVNAA) system to detect and quantify Potassium in small animals. To quantify the expected dose and detection limit that would help set up standards for irradiation, measurement, and calibration in future studies. This system would be adopted accordingly in the future, first for the in vivo studies in large animals, i.e., pigs, and later on human subjects. Here, we demonstrate total potassium measurement in the mouse model (both male and female) with the neutron generator based irradiation assembly. Also, the radiation dose is calculated to ensure safe use. This study provides proof-of-principle for future *in vivo* potassium studies in humans and small animals.

## Results

### Monte Carlo simulation

#### MC simulation model for irradiation assembly

**Figure 1:**
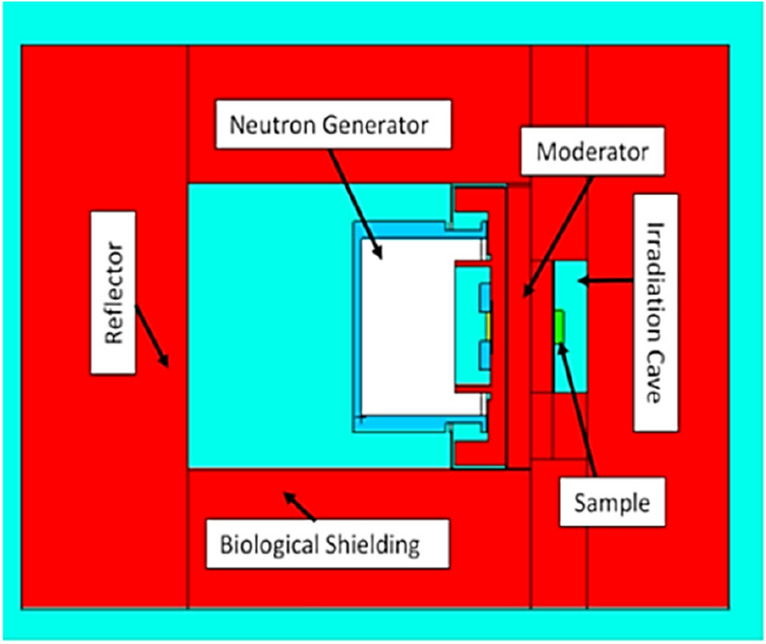
In vivo neutron irradiation assembly along with the components (moderator, reflector, biological shielding) was modeled in MCNP (XY view of the assembly shown here. (created with MCNP 6.1 https://laws.lanl.gov/vhosts/mcnp.lanl.gov/index.shtml).

The in vivo neutron activation analysis assembly was modeled in detail with the Monte Carlo N-Particle (MCNP) software. The optimized simulation model was utilized to simulate the measurements for the phantoms and the mouse samples. Figure 1 shows the simulation model of the neutron activation irradiation facility based on the DD110 neutron generator. Both moderator and reflector in the in vivo neutron activation analysis assembly (IVNAA) were made of polyethylene blocks. Thermal and intermediate neutron flux contributed most to activate the potassium (k-41) nuclei in mice.

#### MC simulation model for HPGe detector and measurement cave

**Figure 2:**
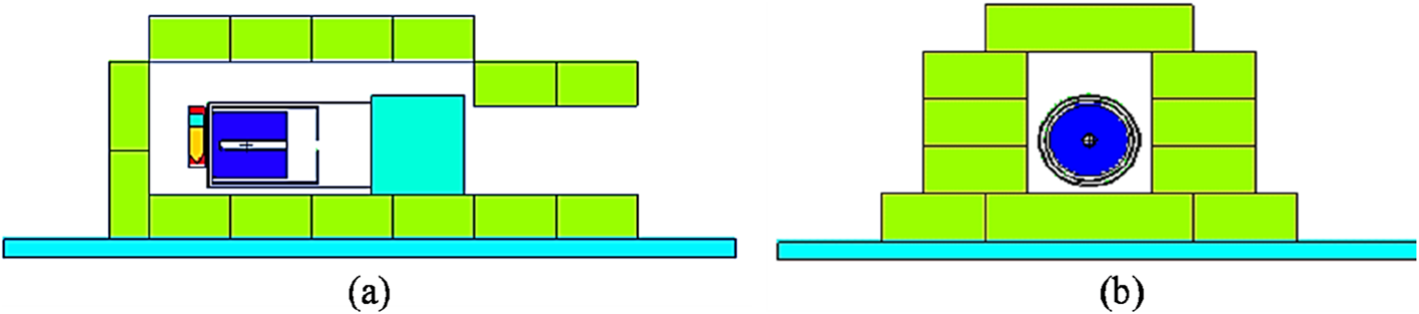
In vivo neutron irradiation assembly along with the components (moderator, reflector, biological shielding) was modeled in MCNP (XY view of the assembly shown here) (created with MCNP 6.1 https://laws.lanl.gov/vhosts/mcnp.lanl.gov/index.shtml).

To estimate the efficiency of the coaxial HPGe detector accurately, MCNP was used to simulate the detector. Figure 2 shows the two cross-sectional views of the model in the MC simulation. It was of interest to develop the experimental facility’s simulation model and benchmark the detection efficiency with the known source and standard geometry. The simulation model is used to estimate the gamma counts from the phantoms and the mouse samples in this project. It would also be useful in future studies, especially for the geometries of the samples that do not follow the standard shapes, e.g., human hand. Most of the HPGe detector dimensions employed in the MCNP model were obtained from the manufacturer. However, dead layer dimensions were readjusted to match the simulation results with the experiment. Simulation and experimental efficiencies for lower energy regions differ 40% or more before adjusting the dead layer. A mixed liquid source consisting of Cd-109, Co-57, Te-123m, Sn-113, Sr-85, Cs-137, Co-60, and Y-88 radionuclide was used to obtain the experimental efficiency. As mice were placed 0 cm from the detector, hence the standard source was also placed at 0 cm from the detector. Table 1 shows the experimental efficiencies and simulation efficiencies (after dead layer adjustment) at 0 cm from the detector surface. The detector efficiency of the K-42 gamma-ray was estimated from the experimental results listed in Table 1.

**Table 1:** Experimental efficiency vs. simulation efficiency of the 100% efficient HPGe detector measured with a multi-nuclei liquid source at 0 cm.

Radionuclide	Energy (keV)	Experimental efficiency	Simulation efficiency	Difference (%)
cd-109	88	1.13E−01	1.11E−01	1.05
c0-57	122	1.30E−01	1.27E−01	2.54
Te-123m	159	1.26E−01	1.20E−01	4.52
Sn-113	392	7.36E−02	7.83E−02	6.41
Sr-85	514	6.22E−02	6.66E−02	7.10
Cs-137	662	5.25E−02	5.62E−02	6.99
Y-88	893	4.37E−02	3.97E−02	8.99
Co-60	1173	3.67E−02	3.49E−02	4.74
co-60	1333	3.43E−02	3.22E−02	6.20
Y-88	1836	2.78E−02	2.46E−02	11.56

**Figure 3:**
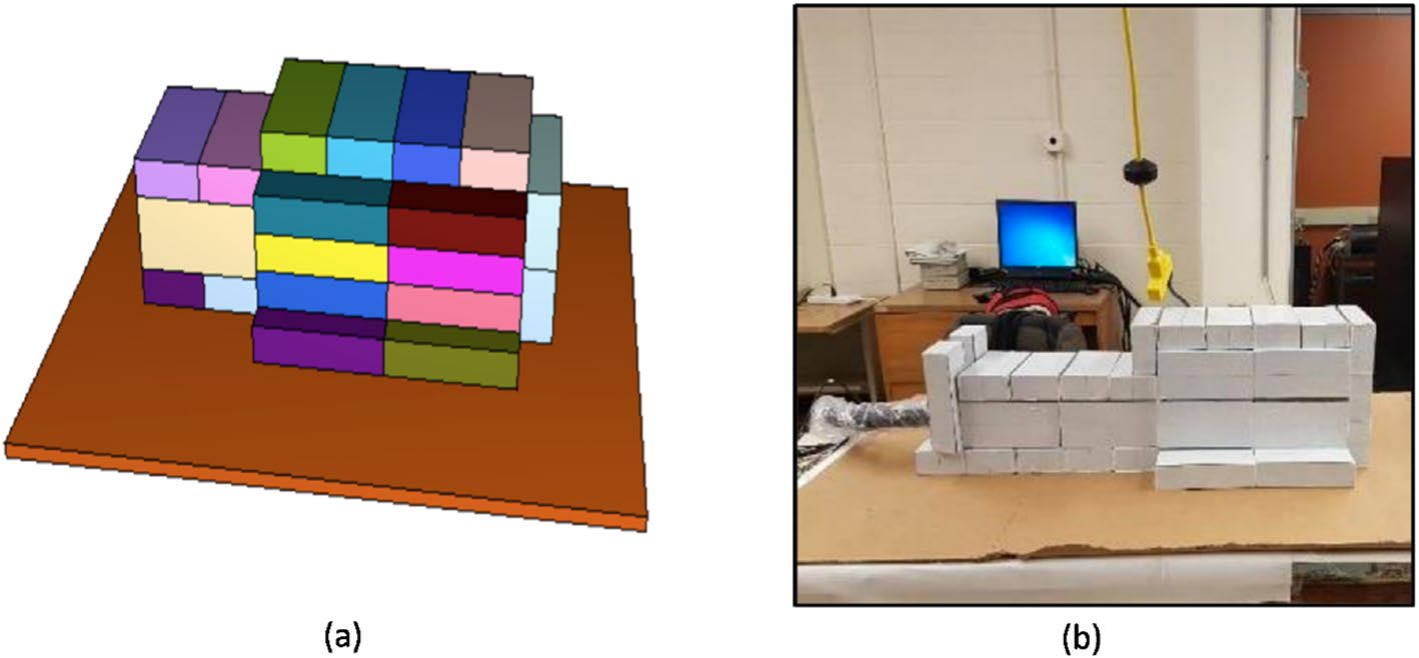
Lead shielding cave built around the HPGE detector to measure gamma counts from the sample after irradiation **(a)** MCNP simulation model of the detector and cave **(b)** lead shielded cave built around the experimental setup (created with MCNP 6.1 https://laws.lanl.gov/vhosts/mcnp.lanl.gov/index.shtml).

### Background reduction with an irradiation cave

**Figure 4:**
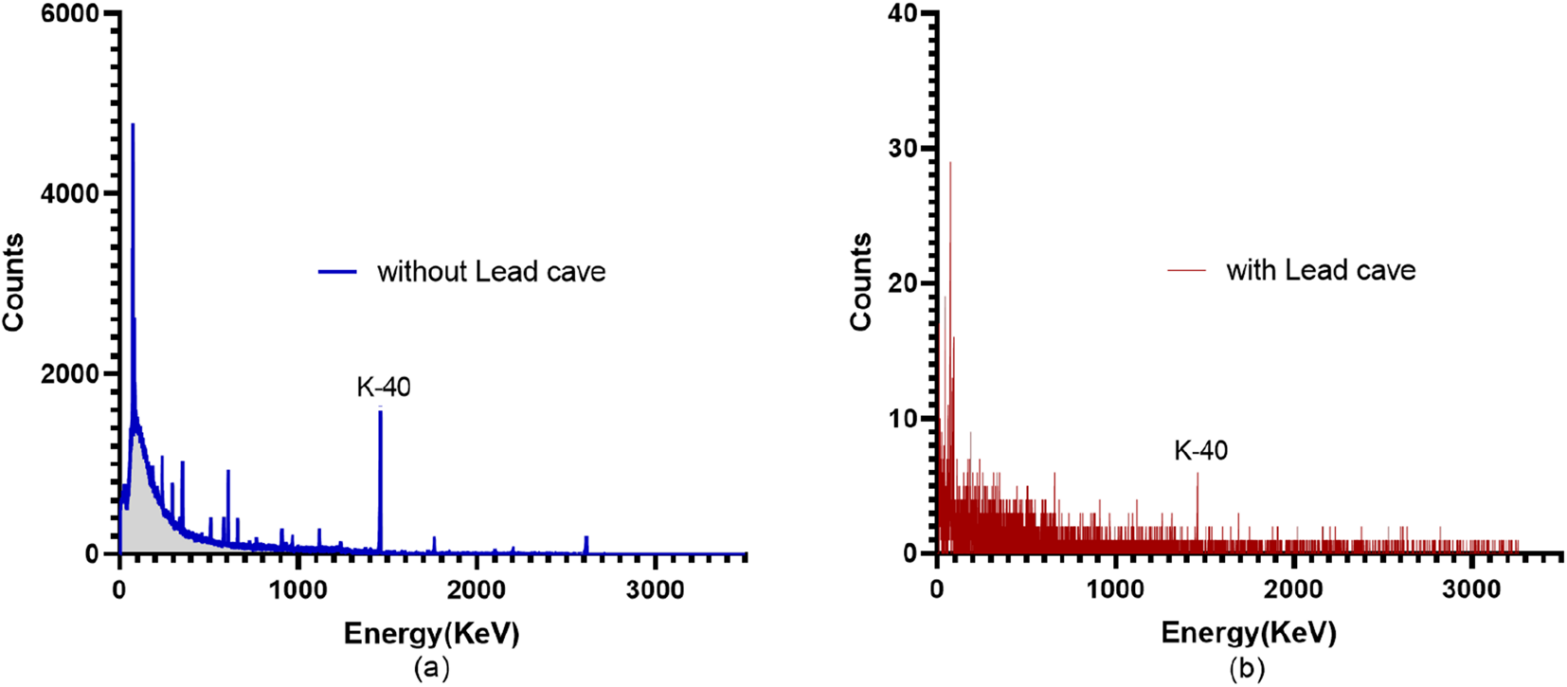
Background radiation spectrum obtained with the HPGe detector without any sample **(a)** without lead cave and **(b)** with lead cave.

For any element of interest, e.g., potassium, background radiation influences the detection limit that can be achieved with the given neutron activation analysis system. To obtain a lower detection limit, we designed a shielding cave around the HPGe detector system. Figure 3 shows the shielded HPGe detector and an equivalent simulation model in the MCNP. As our lab performs in vivo experiments for different subjects, customized shielding caves can be designed accordingly. The shielding cave can have an opening to place the human hand for the /if in vivo hand metal measurement, but it was entirely closed for the ex vivo small animal measurement performed in this study. Figure 4 compares the background radiation obtained, with and without lead shielding cave, for 1-hour measurement. A significant reduction in well-known k-40 characteristic gamma-ray can be seen in the spectra.

### System calibration and detection limit

**Figure 5:**
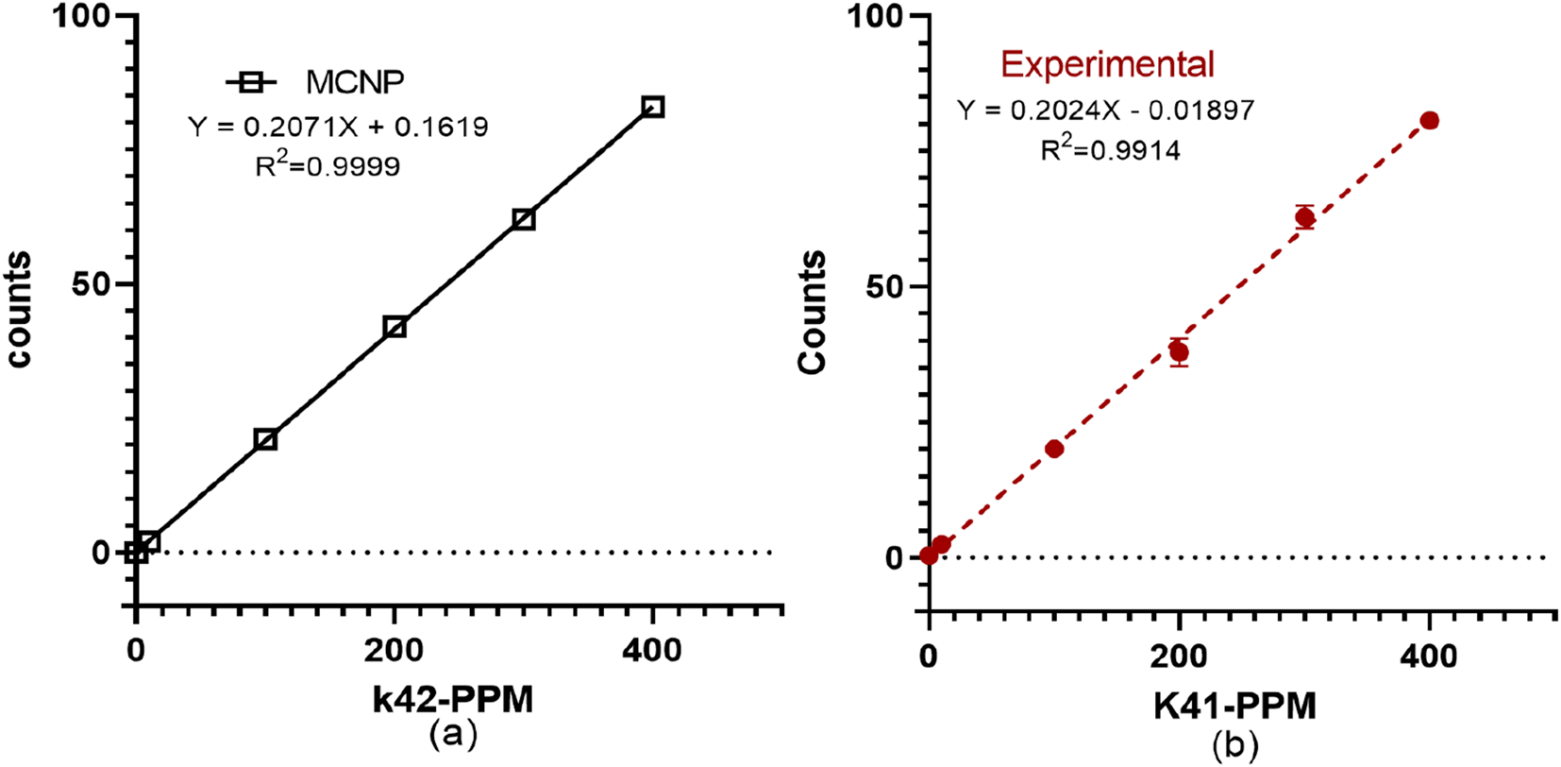
Calibration line for the potassium aliquots of 0 ppm, 10 ppm, 100 ppm, 200 ppm, 300 ppm, 400 ppm **(a)** MCNP simulation by using the modeled irradiation assembly **(b)** phantoms measured with experimental setup.

Potassium doped phantoms were used for the system calibration. Figure 5 shows the calibration lines obtained from the simulations and the experiments. Table 2 shows the K-42 gamma-ray counts calculated from the simulation and the experiments. The simulation results agree well with the experimental results, which further validate the simulation models. We calculated the detection limit by using Eq. (2) shown in method section for the potassium phantoms. The background was estimated under the region of $\pm 2\sigma$ centered at 1525 keV gamma-ray. The detection limit for the potassium concentration for the NAA assembly is calculated to be approximately 46 ppm.

**Table 2:** Counts obtained with 0 ppm, 10 ppm, 100 ppm, 200 ppm, 300 ppm, and 400 ppm doped potassium (41K) phantoms obtained with MCNP simulation and experiment.

Phantoms (ppm)	Counts $T_{irrad}=$ 10 min		Ratio
	Simulation	Experimental	
0	0	1	0
10	2	3	0.72
100	21	20	1.05
200	42	35	1.18
300	62	65	0.96
400	83	80	1.03

**Figure 6:**
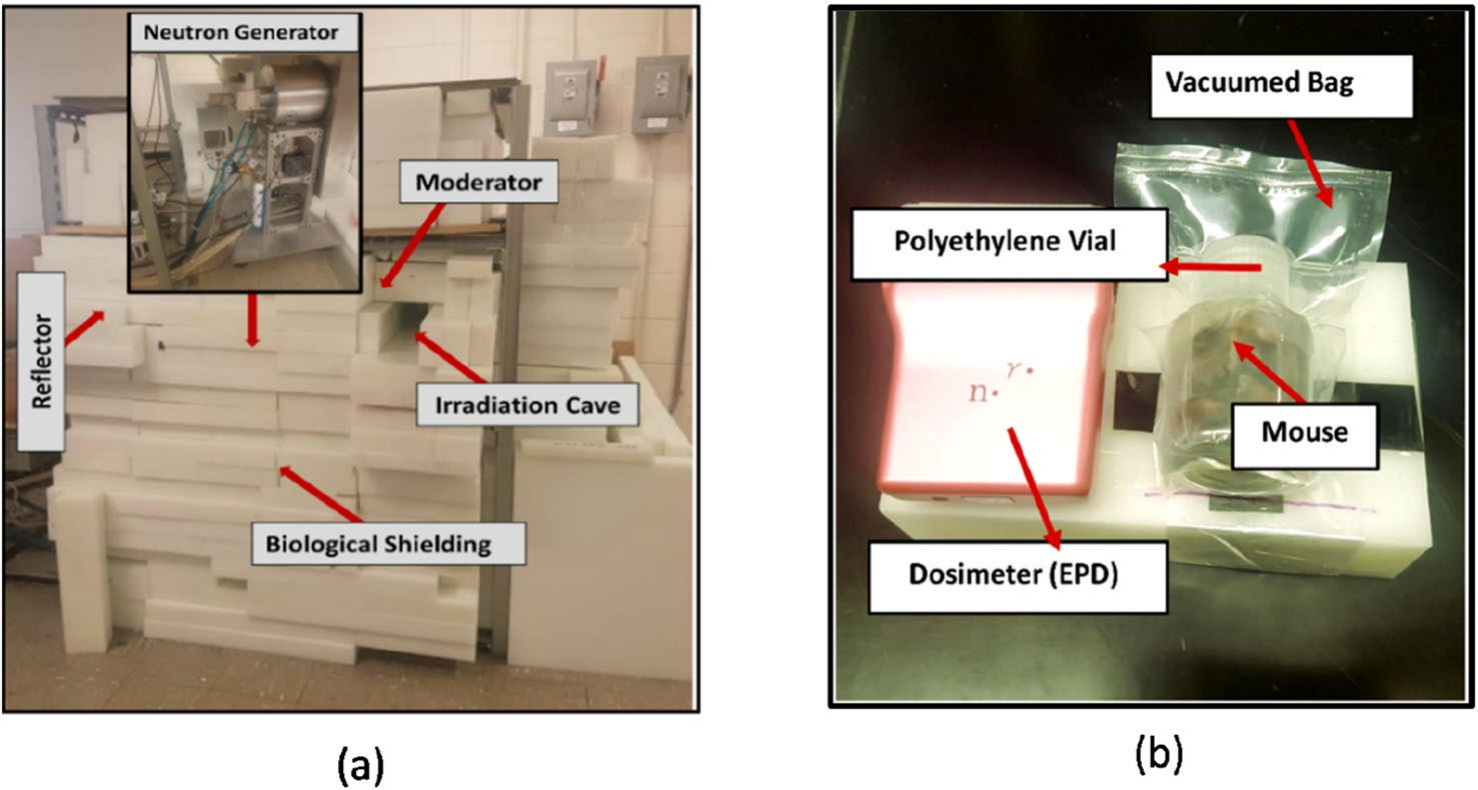
**(a)** The experimental setup made of irradiation assembly based on neutron generator to measure potassium concentration **(b)** mice sample before irradiation. Mice samples were placed inside the irradiation cave shown in **(a)** for the measurement.

**Figure 7:**
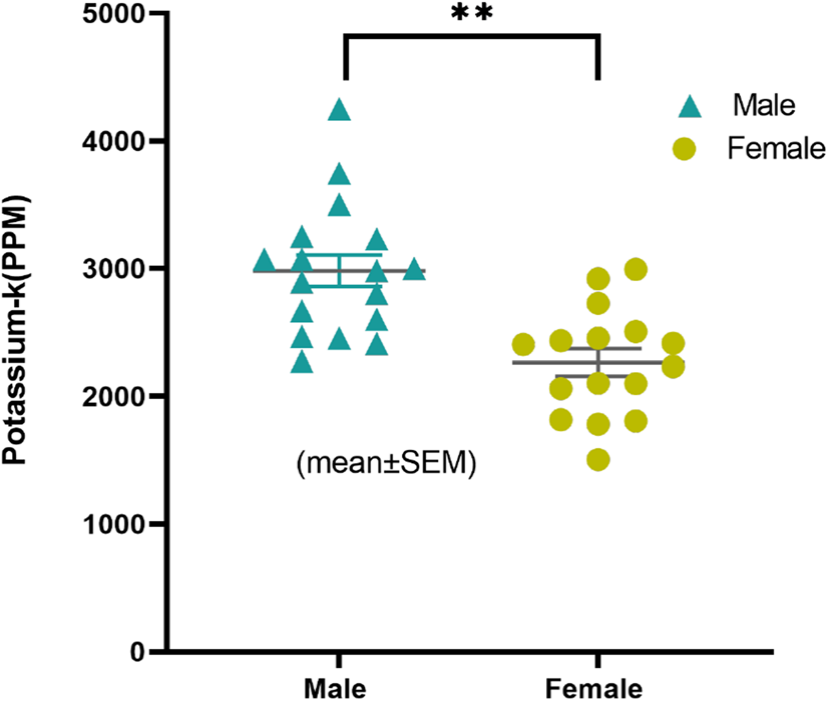
Potassium concentration (mean ± SEM) of male and female mouse samples after 10 min irradiation. Male n $=$ 16; female n $=$ 16. Unpaired two tailed t test (t $=$ 4.35, df $=$ 30); p $=$ 0.0001, significant difference indicated by (**).

**Figure 8:**
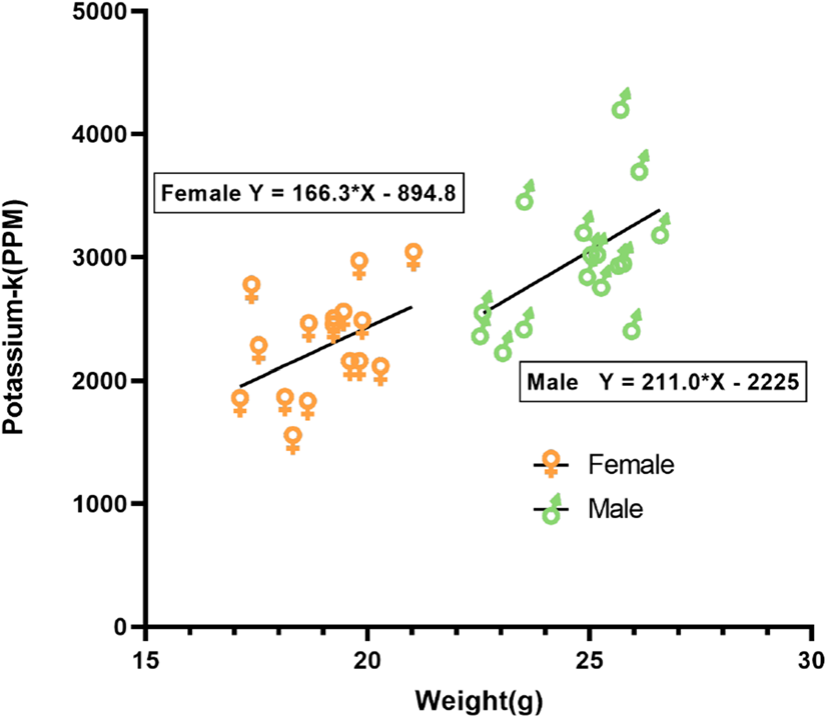
Linear regression performed for potassium concentration in male, female mice with weight, slope for females (166.3 ± 91.38), and male (211.1 ± 92.2) regression lines were not significantly different.

### Mouse measurement and radiation dose

**Figure 9:**
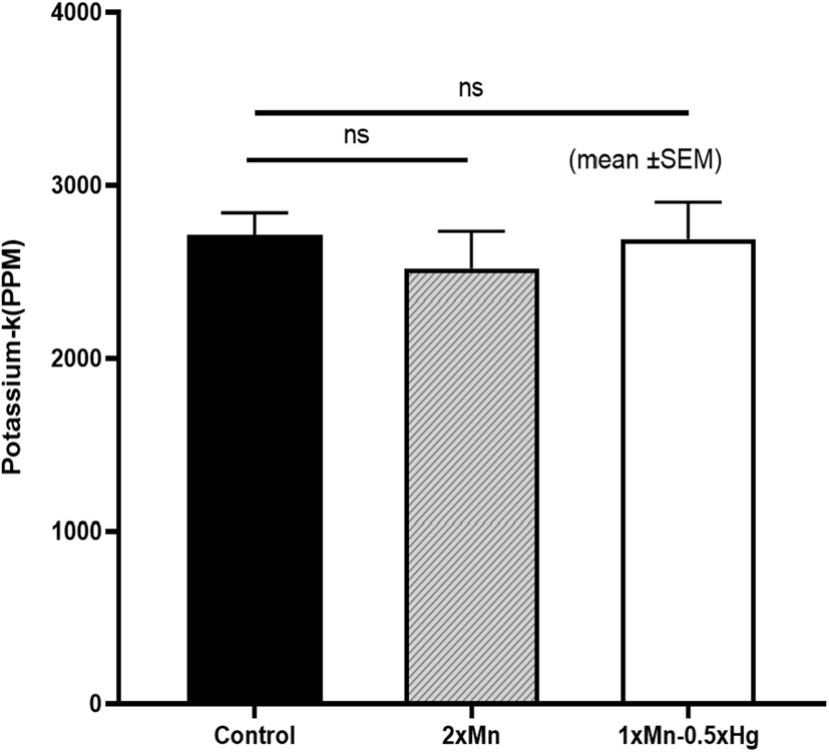
The mean (± SEM) potassium concentration in three groups of mice control, manganese exposed, and mercury exposed (including both male and female), showed non-significant (ns) difference, with P $=$ 0.713.

Figure 6 shows the neutron activation analysis assembly, and a prepared mouse sample. Each mouse sample was placed inside the irradiation cave for 10 minutes, while the cave was entirely closed with polyethylene blocks. All mouse samples were irradiated and measured twice. Table 3 (given on last page) reports the concentrations of total body potassium measured in all 32 mice and the standard deviations. An unpaired two-tail t-test was performed to test whether the male mice and female mice had significantly different mean potassium concentrations (ppm). The normal distribution of potassium concentration in both sexes was verified with the D’Agostino & Pearson test (K2 $=$ 2.8 and 0.155) before conducting a t-test. Additionally, the assumption of homogeneity of variance was tested and satisfied via the F test. The result shows that male mice have a significantly higher mean K concentration than that in female mice (3001 ± 527 vs. 2266 ± 419 ppm; t (30) $=$ 4.35, p $=$ 0.0001). Figure 7 shows the potassium distribution in both male and female groups. These results suggest that the potassium concentration in both sexes was different.The difference in the weight of male and female mice was also observed. Linear regression was plotted to predict potassium concentration based on weight, as shown in Fig. 8. Results of the Pearson correlation indicated that there was a significant association between potassium concentration and weight in male mice and a marginally positive association in female group (for male r(16) $=$ 0.52, P(two tailed) $=$ 0.038; for female r(16) $=$ 0.43, P(two tailed) $=$ 0.09). We also assessed the impact of manganese (Mn) and mercury (Hg) on the potassium concentration. With one-way ANOVA performed on the three groups, i.e., control, mercury exposed group (1$\times$Mn-0.5Hg), and manganese exposed group (2$\times$Mn), the results showed no statistically significant differences between the group means (F (2, 29) $=$ 0.341, P $=$ 0.713). Figure 9 shows the mean potassium concentration in three groups, whereas the bar shows the standard error of the mean. During the experiments, the radiation equivalent dose was found to be approximately 56 mSv inside the irradiation cave, monitored with an electronic pocket dosimeter and TLD. The neutron and photon equivalent doses were found to be 49 mSv and 7 mSv, respectively.

## Discussion

In this study, we found that potassium concentration could be measured within small animals like a mouse, with a DD110 neutron generator based neutron activation analysis system. The detection limit for potassium with the existing IVNAA system was calculated to be 46 ppm approximately. The experiments were performed with the same protocol used earlier for humans; hence, the acquired detection limit with a radiation dose of 56mSv showed the feasibility of potassium measurement in humans (with average tissue level of  2000 ppm) with the IVNAA system. We expect to achieve better experimental accuracy with the human hand ( 350 g human hand vs. 25g mouse). Moreover, the redesigned lead cave reduced the background radiation effectively, ensuing an overall efficient counting system with an improved detection limit. The average potassium concentration was measured as $3001 (\pm 527)$ ppm in male mice and $2266(\pm 419)$ ppm in females. The concentration was slightly higher than  2000 ppm reported in ICRP23 and 2080 ppm in the human soft tissue reported by Christy et al.^30,31^. It is customary in the literature to use the same elemental composition for the mice, as reported for the reference man (ICRP89 & ICRP23)^32^. We compared our results with an absolute concentration of potassium found in mice in the literature for neutron-activation: H. C. Bigginand et al. reported 0.24% average potassium concentration in mice compared with an average of 0.26% (average% by weight) computed in our experiment^26^. Similarly, Nicoletta et al. observed 3000–3600 ppm potassium in mice^33^. Another study by Yannga et al. reported 4490 ppm potassium concentration in the skeletal muscle of the control mice^34^. The higher potassium found in this study could be attributed to the measurement in skeletal muscle alone, as ICRP89 also reported the highest potassium concentration in skeletal muscle (0.4%). The mean potassium concentration in Yannga et al.’s study, contributed by all organs was approximately 3432 ppm. In this study, the mean potassium concentration in the female mice was observed lower than that of the male, consistent with the earlier findings in humans^35,36^. However, no data to the best of our knowledge exists on sex-based potassium concentrations in mouse. Here, the absolute potassium concentration in females was 27% lower than male potassium concentration. However, normalized with skeletal muscle ratio (ICRP89 reported male skeletal muscle percentage approximately 1.5 times higher than female skeletal muscle)^37^ the difference between males and females was < 5%. Weight was marginally positively correlated to the potassium concentration for both male and female mice. The IVNAA assembly should be further optimized to achieve a lower dose and reduced measurement time. The in vivo studies on small animals that address the monitoring of potassium kinetics, the role of dietary potassium on blood pressure, and related cardiac diseases in conjunction with dietary sodium have been critical areas of interest for nutritionists, clinicians, and health policymakers. However, traditional bio-markers like serum and 24 h. Urine samples used in these studies were less tolerated by animals, proved inconvenient, and poorly correlated with total body elemental deposition in some cases. Our study showed that the IVNAA assembly could conveniently and accurately measure potassium with a very low dose; hence, this system is expected to be a highly reliable, economical, and efficient alternative to the traditional measurement methods. This study evaluated the feasibility of potassium measurement in rodents and the IVNAA system’s development to permit the novel potassium studies in animals and humans. One of the limitations of this study is that no physiological data like blood pressure, bone density, etc were presented, which could have helped to understand the impact of potassium intake and retention with health outcomes. Nevertheless, Our next study includes the methodology development to non-invasively quantify the potassium concentration and distribution in soft tissue/muscle and bones. This multi-element technique can simultaneously measure sodium and would be a novel method to monitor the exchangeable potassium and sodium in humans. Future research includes the methodology development to non-invasively quantify the potassium concentration and distribution in soft tissue/muscle and bones. This multi-element technique can simultaneously measure sodium and would be a novel method to monitor the exchangeable potassium and sodium in humans. The IVNAA offers the opportunity to directly observe the potassium kinetics, measure potassium concentration, and determine the potassium’s bone deposition. We believe that IVNAA measurement can facilitate the assessment of dietary nutrient’s role in hypertension, cardiovascular diseases, and bone health. Likewise, tissue potassium measurement in the presence of sodium intervention study in the future could provide new insights to use potassium for disease prevention.

## Conclusion

Our potassium study on the mice demonstrated that the IVNAA system could provide an efficient, accurate, and effective way to measure and monitor potassium concentration in small animals. Our efficient and reliable method of monitoring potassium concentrations, and studying in vivo potassium deposition simultaneously with sodium and other elements could be very useful in understanding the role of potassium in lowering blood pressure and associated diseases.

## Methods

**Figure 10:**
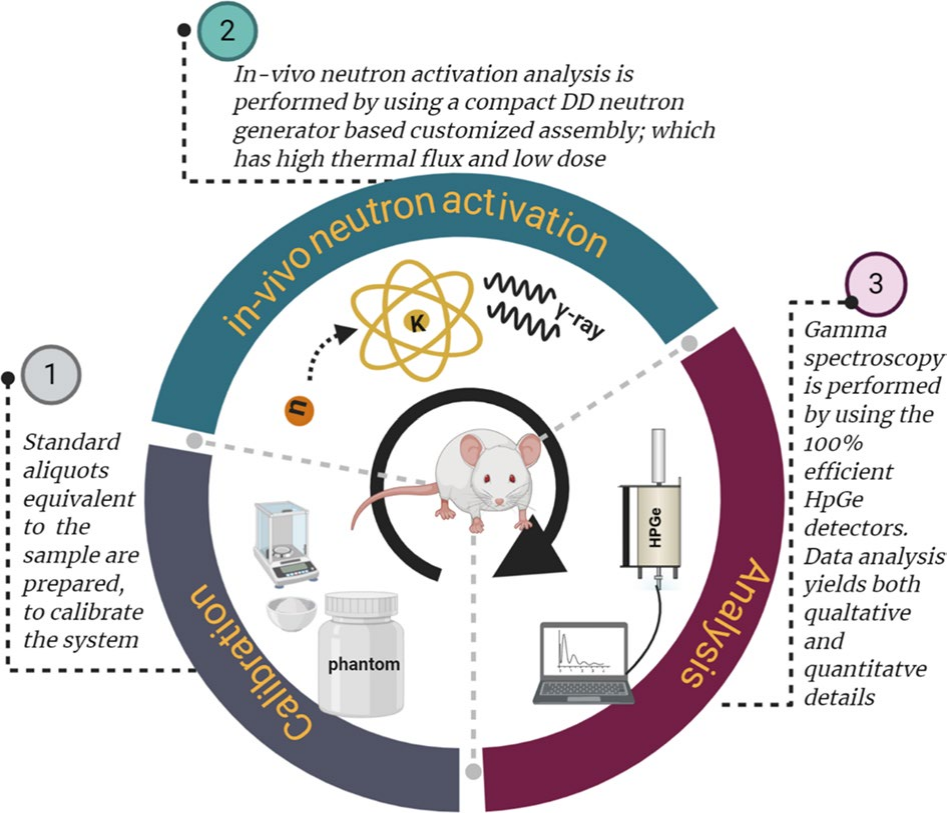
Schematic layout of the steps used for the neutron activation analysis, starting with preparation of standard phantoms for calibration, then with samples placed in the irradiation assembly, and finally with data collected using a high purity germanium system [created with BioRender.com (https://app.biorender.com/)].

In this section, we describe in detail the animal preparation, the simulation and experimental procedures used for the system calibration, measurement of potassium in mice, and the data analysis methods. Figure 10 shows the generalized layout of the neutron activation analysis procedure performed for this study. As we aim to test and prepare the system for the future in vivo measurement of the potassium in humans and small animals, relevant quantities such as dose and detection limit were also calculated.

### Animal preparation

Thirty-two C57BL6/J mice, in two separate cohorts, were used in this study. There were four male and four female mice in the first cohort, whereas the second cohort had 12 male and 12 female mice.The mice were treated and handled according to the IACUC protocol which was reviewed and approved by the “Purdue Animal Care and Use Committee” (PACUC; Protocol number 1809001794). We confirm that all experiments were performed in accordance with relevant guidelines and regulations. Before sacrificing, mice were kept in the specially designed polycarbonate cages to minimize external contamination, where they had free access to the standard mice food AIN-93G. All mice were sacrificed at the age of 20 weeks; later, they were weighed and placed in cylindrical vacuum-sealed polyethylene vials before neutron activation analysis. These polyethylene vials were utilized to achieve consistent geometry and ease of handling. The body parts, i.e., brain, liver, and kidneys were removed from the carcasses used in this study. The total body-weight of mice was monitored regularly before sacrificing, and the final weight of the carcasses was also recorded. The mice were part of another study to investigate biomarkers for manganese (Mn) exposure, where each cohort was further divided into three subgroups, i.e., control group, double manganese (Mn) exposure group (2xMn), and methylmercury (Hg) plus Mn exposure group (1xMn-Hg). 2xMn exposure groups received six doses (on day 0, 3, 6, 20, 23, and 26) of 50 mg/kg of MnCl2.4H2O each day over a period of sixty days (equivalent to a Mn dose of 13.9 mg/kg); the 1xMn.Hg group received Mn only on days 0, 3, and 6). Additionally, 1xMn.Hg exposure group from cohort two received three doses (days 20, 23, and 26) of 1.5 mg/Kg of Hg. As exposure was 1.5 mg/kg of MeHg, so 1.4 mg/kg of Hg alone, respectively was administered.

### Monte Carlo simulation

Monte Carlo N Particle (MCNP) code developed by the Los Alamos laboratory is a reliable code to simulate the transportation of the radiation particles (i.e., neutron, photons, electrons, etc.) in a user-defined model^38^. MCNP used the physics model and evaluated nuclear data libraries to track particle’s interactions and the generation of secondary particles over a broad energy range. We utilized MCNP 6.1 to develop the model of the in vivoneutron activation in our lab.

#### MC simulation model for the irradiation assembly

The in vivo neutron activation assembly (IVNAA) assembly in our lab is based on the DD110 neutron generator. The specific details of the design were described in our previous study^28^. This IVNAA system was explicitly designed to have high thermal flux and low dose for in vivo measurements on humans and small animals. In this study, the MCNP simulation model of the IVNAA was developed to compare the simulation of potassium phantom and mice with experiments. The Monte Carlo (MC) simulations helped to estimate the actual neutron flux and energy spectra, which in turn helps to validate if the experimental assembly is working up to the expectation. The MC simulation method, similar to the experiment, was applied to investigate the feasibility of potassium measurement with the neutron irradiation assembly.

#### Calculation of the gamma-ray counts

We simulated the soft tissue equivalent mouse phantoms. These phantoms were made of water and doped with different amounts of potassium. A simulated calibration line was obtained for the 1525 keV gamma rays obtained via $41K(n,\gamma )42K$ reaction. The simulation followed the same experimental protocol for irradiation and measurement, as described in the section below. The expected K gamma-ray counts were calculated using Eq. (1) for these phantoms.

1 $$\begin{aligned} Np = N_k .R_k .\gamma _k .\varepsilon _k.S_a.D_a.C_a \end{aligned}$$

where Np = gamma-ray counts from the full energy peak of the element, whereas $N_k = \frac{m.\omega .N_A}{M}$ for which m = sample mass (g), M = molar mass (g/mol), $N_A$ = Avogadro’s number $(6.023 \times 10^{23} molecules/mol)$, $\omega$ = isotopic abundance ; $R_k = \phi _e.\sigma _o$ = reaction rate per nucleus; $\gamma _k$ = gamma intensity; $\varepsilon _k$ = detector efficiency; $S_a = (1 - e^{-\lambda t_i} )$ irradiation factor for which $t_i$ = irradiation time; $D_a = decay factor (e^{-\lambda t_d })$ for which $t_d$ = decay time, and $C_a = (1-e^{-\lambda t_c} )/\lambda$ count factor for which $t_c$ = counting time.

#### MC simulation model for HPGe detector and measurement cave

MC simulation was also used to calculate the absolute efficiency of a coaxial p-type high purity germanium (HPGe, model # GMX100P4-95-A) detector. The simulation model was required to characterize the detection system and predict the overall system’s performance before the experiment. In the model, a pulse-height tally (F8) was employed to simulate the detector response; it registered all the pulses produced in the detector’s active volume and presented them as pulse height distribution. The FT8 GEB option was utilized in the model to accommodate the Gaussian energy broadening (GEB) phenomena observed in the experimental spectrum. The GEB card parameters were obtained from the experimental spectrum to match the full-width at half maximum (FWHM) in the experiment. A standard multi-nuclide gamma source (Catalog No. 7500; Eckert & Ziegler Isotope Products, Inc. Valencia, CA), which contains Cd-109, Co-57, Te-123m, Cr-51, Sn-113, Cs-137, Co-60, and Y-88 radionuclide was simulated as a gamma source. The vial of the source resembled in shape with the vials used for the phantoms and mouse samples; hence, it minimized the geometry effects on the detector’s efficiency. The total number of histories in MCNP was chosen to keep the relative error/uncertainty below 3%. The simulated spectrum and absolute efficiencies were matched with the experimental spectrum of the same source.

**Table 3:** Potassium concentration $(mean \pm S.D.)$ measured in n $=$ 32 mice, with IVNAA, in two sets of the irradiation.

n $=$ 16, male			n $=$ 16, female		
Mouse ID #	Weight (g)	K $(\pm$ S.D.) (PPM)	Mouse ID #	Weight (g)	K $(\pm$ S.D.) (PPM)
1	22.59	2601 (±981)	2	19.82	2103.9 (±802)
3	24.95	2893 (±385)	4	21.04	2993.3 (±1196)
5	23.05	2274.8 (±551)	6	17.38	2725.8 (±320)
7	22.54	2410.7 (±220)	8	18.32	1506.3 (±106)
13	24.87	3249.6 (±1166)	14	19.60	2102.4 (±991)
15	25.95	2452.6 (±583)	16	19.81	2918 (±653)
17	25.65	2982.9 (±157)	18	17.55	2233.1 (±209)
19	23.53	3503.2 (±820)	20	19.46	2506 (±236)
21	25.03	3071.4 (±304)	22	20.30	2062.8 (±228)
23	25.18	3071.5 (±411)	24	19.24	2452.7 (±82)
25	25.26	2805.7 (±509)	26	18.65	1783.5 (±305)
27	25.70	4251 (±37)	28	17.12	1807.9 (±79)
29	25.76	3000 (±317)	30	18.68	2413 (±531)
31	26.12	3746.8 (±108)	32	18.14	1817 (±72)
33	26.59	3232.1 (±384)	34	19.88	2434.1 (±583)
35	23.52	2465.5 (±556)	36	19.23	2402.9 (±352)
Mean	22.02	2846.6 (±525)		17.9	2116.2 (±432)

### System set up and calibration, and detection limit

The IVNAA assembly, with a specially designed irradiation cave, was used for the experiment^28^ (Fig. 2). To calibrate the system, we prepared a set of mouse equivalent phantoms. These phantoms were used to develop the calibration line and determine the detection limit of the IVNAA system. As the average mass of the mice is about 25 g, we prepared five aliquots of 25 ml deionized water. These aliquots were doped with 10 ppm, 100 ppm, 200 ppm, 300 ppm, and 400 ppm of k-41, which was obtained from 99.9% pure potassium nitrate (KNH3) solution. Aliquots were prepared with similar vials, as used for the mouse samples, to reduce the geometrical differences between the phantoms and the mouse samples. Each phantom was carefully placed in the irradiation cave to be irradiated for 10 min and then moved to the measurement site. Before the 2 h measurement started, each phantom was decayed for 10 min. For gamma spectroscopy, a high-efficiency HPGe detector (model GMX-100P4-95-A) with the relative efficiency of 100% and resolution (or FWHM) of 2.7 keV at 1332 keV was used. A customizable shielding cave was constructed around the HPGe detector to reduce the natural gamma background. The shielding cave was built with lead bricks (2 ” x 4 ” x 8 ”) and could be altered easily for several different types of measurement. The cave enclosed the detector completely, except a small opening for the electronics and electrical coolant. Characteristic gamma rays of 1525 keV from 42K were measured using the well-shielded measurement system. The detection limit for the potassium concentration in the mice was determined using the slope of the calibration line obtained from the potassium standard phantoms and Eq. (2).

2 $$\begin{aligned} DL= 2*sqrt (bkg)/(counts/\Delta p) \end{aligned}$$

Whereas bkg = background under the characteristic gamma-ray peak, counts = net area under the gamma-ray peak, and $\Delta p$ = known concentration of the element in the phantom in ppm. Here $counts/\Delta p$ is equivalent to the slope of the calibration line.

### Mouse measurement and radiation dose

All mouse samples were placed in the irradiation cave of IVNAA assembly for 10 min, followed by 10-min decay and 2-h of measurement with HPGe detector. After a few days, all mouse samples were irradiated and measured again, with the same procedure, to reduce the experimental variance. Potassium concentration was determined via $41K (n,\gamma ) 42K$ neutron activation and expressed as microgram K per gram mouse (ppm). Neutrons and photons were two primary contributors to the radiation dose in the IVNAA procedure. In this experiment, NRF31 (Fuji Electric Co), an electronic pocket dosimeter (EPD) was placed laterally with the irradiation samples to measure the gamma and neutron dose inside the irradiation cave. We have also measured the dose with TLD dosimeter inside the irradiation cave.

### Statistical analysis

We used Graphpad PRISM V.8.0 (GraphPad Software, Inc., CA, USA)^39^ to perform all statistical analysis tests, where alpha $(\alpha )$ = 0.05 was considered statistically significant. The statistical results were reported as mean $\pm$ standard deviation (SD) or mean ± standard error of the mean (SEM). We evaluated the potassium concentration in both male and female groups with an independent t-test whereas, the Pearson correlation test was conducted to assess the correlation between body weight and potassium concentration. The potential effect of both manganese (Mn) and mercury (Hg) on the potassium concentration was also evaluated with a one way ANOVA (Suppl. Information).

## Supplementary information

Supplementary information.
