# Supplementary material for: Whole body potassium as a biomarker for potassium uptake using a mouse model
Source: Sci Rep. 2021 Mar 18;11:6385. doi: 10.1038/s41598-021-85233-2 (PMC7973570; doi:10.1038/s41598-021-85233-2)
Supplement: Supplementary file 1 — Supplementary information. [file 41598_2021_85233_MOESM1_ESM.pdf]

Whole Body Potassium as a Biomarker for Potassium Uptake Using a Mouse Model

Sana Tabbassum [1,+,*], Pinjing Cheng[1,+], Frank M. Yanko[1], Rekha Balachandran[1], Michael Aschner[2], Aaron B Bowman[1], and Linda H. Nie [1,*]

[1]Purdue University, School of Health Sciences, West Lafayette, 47906, USA
[2]Department of Molecular Pharmacology, Albert Einstein College of Medicine, Bronx, NY, 10461, USA
[*]hnie@purdue.edu; stabbass@purdue.edu
[+]these authors contributed equally to this work

ABSTRACT

Supplementary Material

Two repeated measurements were conducted on each subject to analyze the subject's inherent variation and the variability between measurements, as shown in the box and whiskers plot in figure1 . We used 95% limits of agreement method to

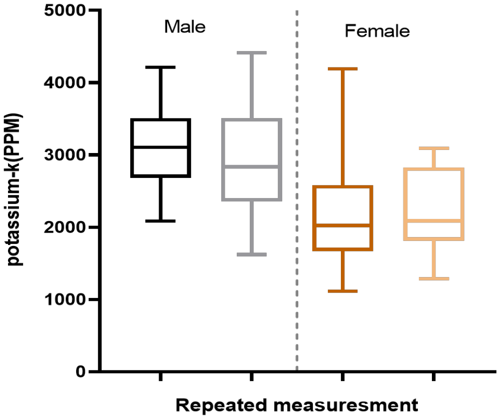

Figure 1. Potassium concentration measured with two successive irradiation performed on male (cohort1+cohort2) and female (cohort1+cohort2) of the mice for variance measurement .

evaluate the reliability of the measurement method[1]. This method helped to quantify the measurement error, i.e., variations between repeated measurements of the same quantity on the same subject. Both assumptions for the 95% limit of agreement method were verified. These assumptions were: a) mean and standard deviation of the differences (between measurement-1 and measurement-2) were constant in the desired range; b) difference between the measurements follows approximately the normal distribution (as shown in the histogram figure 2a). The 95% limit of agreements was ±2100 ppm with 0 mean difference, as shown in the scatter plot figure 2b.

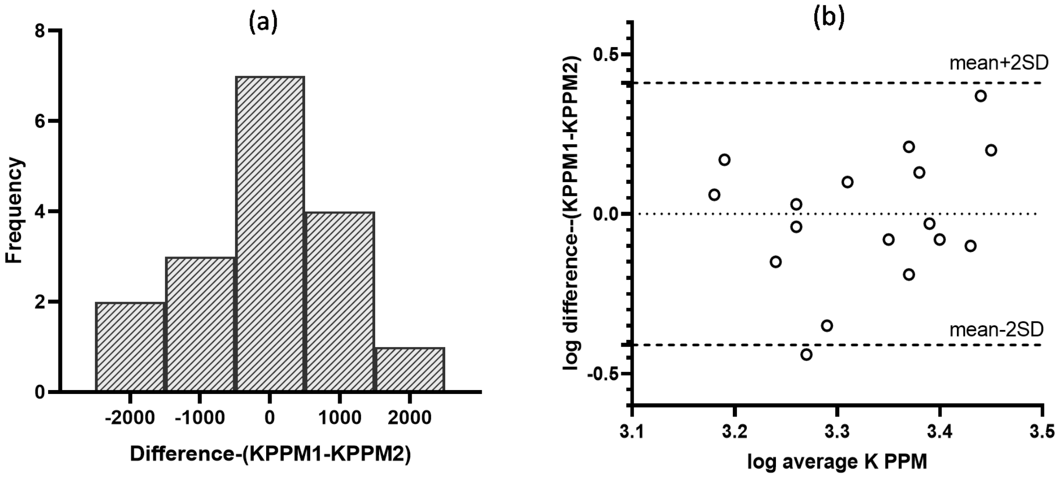

Figure 2. (a) histogram of the difference (b) Plot of log difference against log mean potassium concentration, for the mouse samples

Total body effective dose for an irradiated human hand

Given that, a single hand of the subject is irradiated with thermalized neutron for 10 min resulting in 56mSv equivalent dose. Whereas the mass of hands and arms being about 5% of total body mass. According to ICRP103, the tissue weighting factor for skin and bone is 0.01 (hand and arm is primarily yellow marrow), the effective whole-body dose from hand irradiation can be calculated as

$$E_{eff-h} = Eq_d \times T_w f$$

$$Whereas E_{eff-h} = effective\ dose; \quad Eq_d = equivalent\ dose\ \ and\ \ T_{wf} = Tissue - weighting\ factor$$

$$= (0.05) \times [(0.01 \times 56) + (0.01 \times 56)]$$

$$= 0.056 mSv$$

The external dose to the rest of the body calculated at 0.028 mSv should be weighted to the remaining 95% of body mass, which will give rise to the effective dose as calculated below.

$$E_{eff-RB} = 0.95 \times 0.028$$

$$= 0.026 mSv$$

Whereas E_{eff-RB}=effective dose from rest of body; Total body effective dose would be

$$TB_{eff} = 0.026 + 0.056$$

$$= 0.082 mSv$$

This total effective dose is less than one would receive from one AP chest x-rays (0.1 mSv). Both stochastic and deterministic radiation effects from this low dose are negligible.

References

1. Bland, J. M. & Altman, D. G. Applying the right statistics: analyses of measurement studies. *Ultrasound Obstet. Gynecol. The Off. J. Int. Soc. Ultrasound Obstet. Gynecol.* **22**, 85–93 (2003).
